# Hypoxia-Inducible Factor 1 Is an Inductor of Transcription Factor Activating Protein 2 Epsilon Expression during Chondrogenic Differentiation

**DOI:** 10.1155/2015/380590

**Published:** 2015-07-27

**Authors:** Stephan Niebler, Peter Angele, Richard Kujat, Anja K. Bosserhoff

**Affiliations:** ^1^Institute of Biochemistry, University of Erlangen-Nuremberg, Fahrstrasse 17, 91054 Erlangen, Germany; ^2^Institute of Pathology, University of Regensburg, Franz-Josef-Strauß-Allee 11, 93053 Regensburg, Germany; ^3^Department of Trauma Surgery, University Hospital Regensburg, Franz-Josef-Strauß-Allee 11, 93053 Regensburg, Germany

## Abstract

The transcription factor AP-2*ε* (activating enhancer-binding protein epsilon) is expressed in cartilage of humans and mice. However, knowledge about regulatory mechanisms influencing AP-2*ε* expression is limited. Using quantitative real time PCR, we detected a significant increase in AP-2*ε* mRNA expression comparing initial and late stages of chondrogenic differentiation processes* in vitro* and* in vivo*. Interestingly, in these samples the expression pattern of the prominent hypoxia marker gene* angiopoietin-like 4 (Angptl4)* strongly correlated with that of* AP-2ε* suggesting that hypoxia might represent an external regulator of AP-2*ε* expression in mammals. In order to show this, experiments directly targeting the activity of hypoxia-inducible factor-1 (HIF1), the complex mediating responses to oxygen deprivation, were performed. While the HIF1-activating compounds 2,2′-dipyridyl and desferrioxamine resulted in significantly enhanced mRNA concentration of AP-2*ε*, siRNA against HIF1*α* led to a significantly reduced expression rate of* AP-2ε*. Additionally, we detected a significant upregulation of the AP-2*ε* mRNA level after oxygen deprivation. In sum, these different experimental approaches revealed a novel role for the HIF1 complex in the regulation of the* AP-2ε* gene in cartilaginous cells and underlined the important role of hypoxia as an important external regulatory stimulus during chondrogenic differentiation modulating the expression of downstream transcription factors.

## 1. **Introduction**

Limb morphogenesis in vertebrates is a complex multistep process that starts during embryogenesis and is completed in adults when longitudinal growth of the long bones stops. Initially, multipotent mesenchymal stem cells (MSC) derived from the lateral-plate mesoderm aggregate to form condensations at regions that prefigure the future limb skeleton [1, 2]. After a series of differentiation events limb buds are formed harboring chondrocytes that produce an abundance of extracellular matrix proteins. Hence, an entirely cartilaginous primary skeleton is assembled which grows rapidly and subsequently is replaced by bone tissue during fetal and postnatal development by a progress termed endochondral ossification [3, 4].

Over the years, numerous transcription factors influencing cartilage development were discovered, with SRY- (sex-determining region Y-) box 9 protein (SOX9) and runt related transcription factor 2 (RUNX2) representing master regulators of chondrogenic differentiation [5]. Then again, expression of these intrinsic transcription factors needs to be somehow controlled and tightly synchronized by extrinsic stimuli.

Hormones and growth factors that influence chondrogenesis at various stages play a major role in this process. Of those, Wnt ligands, Indian Hedgehog (IHH), and members of the transforming growth factor-beta family (TGF-*β*) are most commonly described [4–6]. However, more recently, the importance of other extracellular signals affecting cartilage morphogenesis in the fetal limb has been determined. A number of studies suggest that hypoxia is a crucial external stimulus for chondrogenesis and limb development. The primary mediator of adaptive responses of cells to changes in oxygen supply is the transcription factor complex hypoxia-inducible factor 1 (HIF1). HIF1 is a heterodimer that consists of the stable subunit HIF1*β* and HIF1*α*, whose stability is tightly regulated by the intracellular oxygen concentration [7, 8]. Under normoxia, HIF1*α* is hydroxylated by O_2_- and Fe_2_-dependent prolyl hydroxylases (PHD) and quickly degraded [9, 10]. Conversely, under hypoxic conditions these enzymes are inactive resulting in HIF1*α* stabilization and accumulation in the cytoplasm. Subsequently, HIF1*α* translocates into the nucleus and forms the transcriptionally active HIF1 complex after dimerization with HIF1*β*, which then binds to hypoxia responsive elements (HREs; 5′-NCGTG-3′) within the promoter region of target genes and activates their expression [11]. Regarding embryogenesis, Schipani revealed that cartilage structures in the limbs of E15.5 mouse embryos are highly hypoxic and that HIF1*α* is essential for accurate growth-plate and joint formation [12]. This was further addressed by Amarilio et al. who provided evidence that HIF1*α* directly affects early chondrogenic differentiation in the limb bud mesenchyme modulating expression of* Sox9* [13]. The absolute prerequisite of the transcription factor complex HIF1 for proper chondrocyte function becomes clear in mice with an inactivation of* HIF1α* in all cartilaginous structures. They die shortly after birth exhibiting massive cell death in cartilage [14].

Recently, expression of another transcription factor, AP-2*ε*, in human and murine cartilage was revealed by studies in our group [15, 16]. AP-2*ε* belongs to the AP-2 (activating enhancer-binding protein-2) transcription factor family that consists of five isoforms (AP-2*α* to *ε*) and was the last identified member of the group [17, 18]. The proteins are transcriptionally active as homo- or heterodimers and regulate a large number of physiological processes ranging from development and differentiation to tumorigenesis [19]. Other members of the AP-2 family are also known to be expressed during chondrogenesis. For instance, AP-2*α* has an inhibitory function during early chondroblast maturation [20]. Concerning AP-2*ε*, an induction of AP-2*ε* mRNA expression during late stages of chondrogenic differentiation of hMSC in 3D culture was determined [15]. Additionally, we were able to detect the transcription factor in murine hypertrophic chondrocytes as well as in human articular cartilage via immunohistochemistry [15, 16]. Furthermore, AP-2*ε* was shown to be upregulated in osteoarthritic articular chondrocytes resulting in enhanced expression of the chemokine CXCL1 (C-X-C motif ligand 1), which in turn promotes calcification and ECM degradation [21]. Finally, we were able to prove positive regulation of* AP-2ε* gene expression in human chondrosarcoma cells by the transcription factor SOX9 via direct interaction with a consensus binding site within the proximal promoter region of* AP-2ε* [15].

The aim of this study was to further enhance our understanding of regulatory mechanisms modulating AP-2*ε* expression during the chondrogenic differentiation process. Here, special emphasis was put on the analysis of the effects of hypoxic stimuli on the expression rate of the transcription factor in cartilaginous cells.

## 2. **Methods**

### 2.1. Tissue Preparation and Cell Culture

All mouse tissue preparations were carried out under aseptic conditions using SV/129 wild type (WT) mice. Mice were bred at 26°C, 70% relative humidity, and a 12 h light/12 h dark cycle at the university hospital of Regensburg. They were fed with a breeding/maintenance diet (Altromin GmbH, Lage, Germany) and water* ad libitum*. The mice were randomly housed in polypropylene cages with sawdust bedding. The cages were sanitized twice weekly. Animal care and all experimental procedures were carried out in accordance with guidelines of the German law governing animal care. All adult mice (including pregnant females) were killed after anesthetizing by Isoflurane-inhalation (2-chloro-2-(difluoromethoxy)-1,1,1-trifluoro-ethane) via cervical dislocation. New born mice at the age of 4 days were killed by decapitation. According to the German Animal Welfare Act 2006 (article 4) it is sufficient to obtain supervision from the local animal welfare officer (Dr. Thilo Spruss, University Hospital Regensburg) for the killing of mice for scientific purposes (including tissue, embryo, and cell extraction) if no experimental procedures were carried out with the animals. As that was the case in this study, no further notification or approval by the Ethics Committee for Animal Research of the Bavarian government was necessary.

To obtain mesenchymal limb bud cells of E11.5 mouse embryos adult pubescent mice were coupled overnight and the weight of the females was documented. 11 days later, successful pregnancy was determined by a weight increase of at least 3 g. Then, pregnant mice were killed (see above) and embryos were harvested by carefully opening the abdominal wall and the uterus. Subsequently, the embryos were killed by decapitation. All limb buds derived from an entire litter (12 to 32 limb buds each) of a pregnant female were pooled and dissolved in DMEM/F12 (PAA, Pasching, Austria) containing dispase (1 U/mL) (Life Technologies, Carlsbad, California, USA), 10% fetal calf serum (FCS; PAN Biotech GmbH, Aidenbach, Germany), and penicillin (100 U/mL) and streptomycin (10 *μ*g/mL) (both Sigma, Deisenhofen, Germany) at 37°C for 30 min. Single cells were collected by passaging through a 40 *μ*m filter and centrifugation at 280 g for 4 minutes. To refine mesenchymal cells the cells were resuspended in DMEM/F12 (1 : 1) containing 10% FCS and penicillin/streptomycin and incubated for 3 hours at 37°C and 5% CO_2_ for cell attachment. Subsequently, the cells were carefully washed with PBS. This way, cells which do not adhere under these conditions (e.g., endothelial cells) and cells which should rapidly enter apoptosis (e.g., neurons) were removed. After that, the mesenchymal cells were harvested and total RNA was isolated. This is an established procedure to isolate and concentrate mesenchymal cells from murine limb buds, for example, to initiate mesenchymal micromass cultures [22–24]. Overall, the litters of four female mice were utilized.

Epiphyseal cartilage of 4-5-day-old newborn mice was isolated by cutting off the cartilaginous portions of distal femora and proximal tibiae. For each mouse the samples of both hind legs were pooled and total RNA was isolated after pulverization of the cartilage using liquid nitrogen and a mortar. For each time point nine young mice derived from 3 independent litters were used.

Murine MSC were isolated from bone marrow of tibiae and femora of 6–8-week-old mice and cultured as previously described [25]. For the experiments cells of two independent isolations (6 mice each) at passage two to five were used.

Human bone marrow was obtained from the iliac crest of patients undergoing surgery with approval of the Ethics Committee of the University Medical Center of Regensburg, Regensburg, Germany. Written consent from the donors was obtained for use of this bone marrow samples in research. After Percoll gradient fractionation, Dulbecco's modified Eagle's Medium with 10% fetal bovine serum was added to the aspirate and 10 × 10^6^ nucleated cells/100 mm dish were plated and grown at 37°C with 5% CO_2_ until the cells reached 80% confluency. For spheroid formation, adherent cell colonies were trypsinized and counted and 2 × 10^5^ cell aliquots were spun down in V-bottom polypropylene 96-well plates (Nunc, Wiesbaden, Germany) in a defined medium, previously shown to induce the chondrogenic differentiation of these cells in this culture system [26, 27]. The aggregates were then kept in culture up to 28 days for chondrogenic differentiation with medium exchanges carried out every 3 days.

The human chondrosarcoma cell line SW1353 was obtained from the American Type Culture Collection (ATCC, #HTB-94). The cells were maintained in DMEM high-glucose (PAA) supplemented with penicillin (400 U/mL), streptomycin (50 *μ*g/mL) (both Sigma), and 10% FCS (PAN Biotech GmbH) and were incubated in humidified atmosphere containing 21% O_2_, 8% CO_2_ at 37°C. Cells were passaged using trypsin-EDTA solution (Life Technologies) at a 1 : 8 ratio every 3-4 days.

### 2.2. Induction of the Transcriptional Activity of the HIF1 Complex with DP, DFX, or 1% O_**2**_

For treatment of mMSC (murine mesenchymal stem cells) with the hypoxia-mimicking iron chelators 2,2′-dipyridyl (DP; dissolved in EtOH) and desferrioxamine (DFX; dissolved in water) (both Sigma) cells were cultured in six-well plates for 24 h to a density of about 60%. After that, the culture medium was replaced and DP/DFX was added at a concentration of 100 and 250 *μ*M. Respective controls were treated with solvent only. 24 h later cells were harvested and total RNA or protein was isolated. Before incubation of mMSC at hypoxic conditions (1% O_2_) cells were cultured in six-well plates for 24 h to a density of about 60%. After that, the culture medium was replaced and the cells were transferred into a New Brunswick Galaxy 48 series incubator (Eppendorf AG, Hamburg, Germany) flushed with 1% O_2_ and 8% CO_2_. Excessive O_2_ was replaced by nitrogen. Respective controls were incubated at normoxia (21% O_2_). After 24 h cells were harvested and total RNA or protein was isolated. Both experiments were repeated at least five times.

### 2.3. Transient Transfection and Luciferase Assay

siRNA transfection of SW1353 cells was performed using Lipofectamine 2000 (Life Technologies) according to the manufacturer's instructions. Cells were cultured in six-well plates for 24 h to a density of about 40% and transfected with 100 pmol of HIF1*α* siRNA (Hs_HIF1A_5 and Hs_HIF1A_6, Qiagen, Hilden, Germany) or a scrambled negative control siRNA (Qiagen), respectively. Two days after transfection, cells were harvested for RNA or protein isolation. Each experiment was carried out at least three times. For luciferase assays cells were used one day after transfection as described below.

Plasmid-DNA transfection of SW1353 and murine mesenchymal stem cells was performed using Lipofectamine LTX (Life Technologies) as suggested by the manufacturer. For luciferase assays cells were cultured in six-well plates for 24 h to a density of about 60%. Each cationic lipid/plasmid DNA suspension was prepared using 0.5 *μ*g of luciferase reporter plasmid and 0.1 *μ*g of the pRL-TK* Renilla* luciferase control vector in the transfection solutions. 24 h later cells were harvested, lysed, and analyzed for luciferase activity with a luminometer, using Promega dual-luciferase assay reagent (Promega Corporation, Madison, WI, USA). Transfection efficiency was normalized to* Renilla* luciferase activity. To confirm successful treatment of cells with DP, DFX, and HIF1*α* siRNA leading to a modulation of the transcriptional activity of the HIF1 protein complex, a reporter plasmid containing six consecutive HRE binding motifs from the human phosphoglycerate kinase promoter was used (6xHRE; kindly provided by Christina Warnecke) [28]. Additionally, a 604 bp human* AP-2ε* promoter construct was used (AP-2prom604) [15]. For both, promoter activity was normalized to the respective control vector pGL3basic. Each experiment was carried out at least three times.

### 2.4. RNA Isolation, Reverse Transcription (RT), and Quantitative Real-Time PCR (qRT-PCR)

Total RNA of cells and tissues was isolated using e.Z.N.A. MicroElute Total RNA Kit (peqlab Biotechnologie GmbH, Erlangen, Germany) as described by the manufacturers. For isolation of RNA from the pulverized cartilage samples lysis in TRK buffer supplemented with 2%  *β*-mercaptoethanol was expanded to 45 min on ice. Purity as well as concentration was measured in a NanoDrop (peqlab Biotechnologie GmbH). cDNA was generated by RT. The RT reaction was performed in 20 *μ*L reaction volume containing at least 150 ng of total RNA, 4 *μ*L of 5x first-strand buffer, 2 *μ*L of 0.1 M DTT (both Life Technologies), 1 *μ*L of dN6 primer (10 mM) (Roche Applied Science, Mannheim, Germany), and 1 *μ*L of dNTPs (10 mM) (Amersham Pharmacia biotech, Pittsburgh, PA, USA). The reaction mix was incubated for 5 min at 70°C and 1 *μ*L of Superscript II reverse transcriptase (Life Technologies) was added subsequently. RNA was transcribed for 1 h at 37°C. Finally, reverse transcriptase was inactivated at 70°C for 10 min, and RNA was degraded by digestion with 1 *μ*L RNase A (10 mg/mL) (Roche) at 37°C for 30 min.

Quantitative RT-PCR was carried out with the Lightcycler480 system from Roche. A volume of 1 *μ*L cDNA template, 0.5 *μ*L of forward and reverse primer (20 mM), 10 *μ*L of SYBR-Green Premix (Roche), and 8 *μ*L water were combined to a total volume of 20 *μ*L. PCR primers were obtained from Sigma (Table 1). The following PCR program was used: 95°C for 10 min (initial denaturation); 4.4°C sec^−1^ temperature transition rate up to 95°C for 10 sec; 60°C for 10 sec; 72°C for 20 sec, 80°C acquisition mode single, repeated for 45 times (amplification). The PCR product was evaluated by melting-curve analysis. Each sample was analyzed in duplicate. The expression level of the analyzed genes was normalized to the expression level of the house keeping gene*β-actin*.

### 2.5. Protein Isolation and Western Blot

For HIF1*α* protein isolation cells were harvested and treated with a special lysis buffer containing 6.65 M urea, 10% glycerol, 1% SDS, 10 mM tris (pH 6.8), 5 mM DTT, and protease inhibitors (Roche) for 15 min on ice. To shear genomic DNA samples were sonicated in an ultrasonic water bath for 30 sec. After that cell debris was separated via centrifugation at 16,000 g at 4°C for 15 min. Protein concentration was determined using the Pierce BCA Protein Assay Kit (Thermo Fisher Scientific, Rockford, USA). Before measuring, samples were diluted 1 : 3 with water because the kit is not suitable for solutions containing more than 3 M urea.

For SDS-PAGE equal amounts of protein (always 50 *μ*g per lane) were denatured at 70°C for 10 min after addition of Roti-load-buffer (Roth, Karlsruhe, Germany) and subsequently separated on an 8.75% polyacrylamide gel. After blotting onto a PVDF-membrane (Bio-Rad, Richmond, CA, USA) and blocking for 1 h with 5% milk powder in TBS the membrane was incubated overnight at 4°C with a specific primary anti-HIF1*α* antibody (Novus Biologicals, Littleton, USA). After three washing steps with TBS the membrane was incubated for 1 h with an alkaline phosphate-coupled secondary anti-rabbit IgG antibody (Chemicon, Hofheim, Germany) and then washed again. After that, immunoreactions were visualized by BCIP/NBT (Sigma) staining. Subsequently, the membrane was incubated with a primary anti-*β*-actin antibody (Sigma) for 1 h, washed, incubated with an alkaline phosphate-coupled secondary anti-mouse IgG antibody (Chemicon) for 1 h, and washed again. Finally, *β*-actin specific immunoreactions were visualized by BCIP/NBT staining. It should be noted that the anti-HIF1*α* antibody produced one unspecific band which can be detected in all controls but whose intensity was not altered in our experiments. After DP/DFX treatment and under 1% O_2_ the specific band of HIF1*α* became apparent in mMSC (cf. Figures 3(b) and 5(b)). This band was already detectable in SW1353 cells under physiological conditions and its intensity was clearly reduced after transfection of siRNA against HIF1*α* (cf. Figures 4(a) and 4(c)II). Densitometry was performed with the program ImageJ (http://rsbweb.nih.gov/ij/). For the measurement the aforementioned unspecific lower band was excluded.

### 2.6. Statistical Analysis

Results are expressed as mean ± SEM. Comparison between groups was made using the Student paired *t*-test. A *p* value < 0.05 was considered statistically significant (^*∗*^). A *p* value < 0.01 is depicted with two stars (^*∗∗*^) and a *p* value < 0.001 is depicted with three stars (^*∗∗∗*^). All calculations were performed using the GraphPad Prism software (GraphPad software Inc., San Diego, USA).

## 3. **Results**

In a previous study, we revealed a strong induction of AP-2*ε* mRNA expression during late stages of chondrogenic differentiation of hMSC in 3D spheroid culture systems [15]. In the same study, we were able to determine that the transcription factor SOX9 transactivates AP-2*ε* expression in human SW1353 chondrosarcoma cells. However, so far we did not analyze whether there are other modulators of* AP-2ε* gene expression besides SOX9.

**Figure 1:**
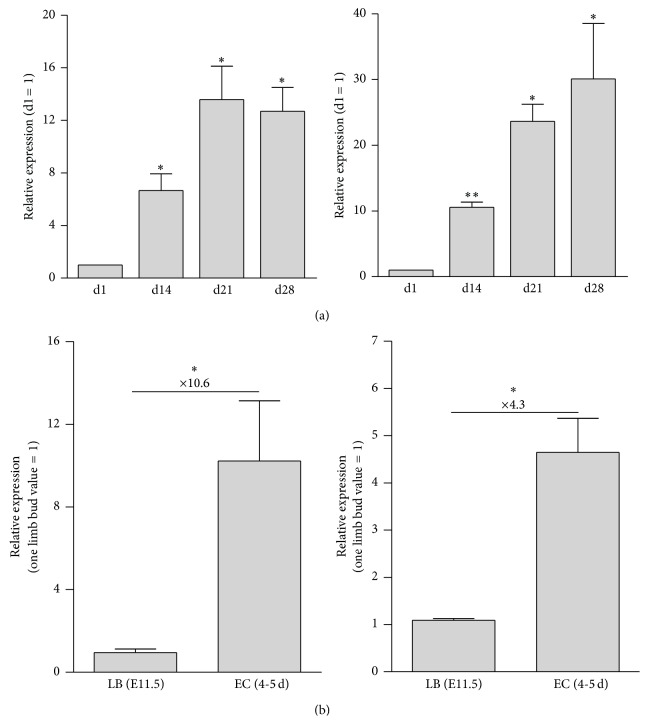
Expression of AP-2*ε* and the hypoxia marker angiopoietin-like 4 strongly correlate during chondrogenic differentiation. (a) 3D spheroid cultures of hMSC were cultured in chondrogenic medium for a period of 28 days and mRNA was isolated at days 1, 14, 21, and 28. Expression of both AP-2*ε* and ANGPTL4 significantly increased over the course of the experiment. (b) mRNA expression was analyzed in mesenchymal cells derived from the limb buds (LB) of E11.5 mouse embryos and in chondrocytes from the epiphyseal cartilage (EC) of 4-day-old new born mice, representing early and late stages of chondrogenic differentiation. In the latter, a significant upregulation of the AP-2*ε* as well as the Angptl4 mRNA level could be detected (data are given as means ± SEM; ns, not significant; ^*∗*^ *p* < 0.05; ^*∗∗*^ *p* < 0.01).

**Figure 2:**
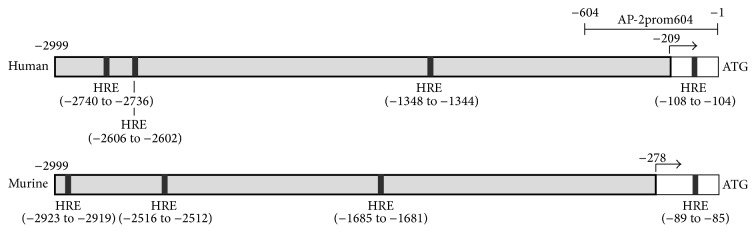
Analysis of human and murine AP-2*ε* promoter sequences for HRE motives. Schematic drawings of the human and murine AP-2*ε* promoter (−2999 to −1 bp). All numbers refer to the translation start. The transcription start is marked with an arrow. Putative hypoxia responsive elements (5′-NCGTG-3′) are depicted as black boxes (HRE). Further, the position of a 604 bp human AP-2*ε* promoter construct (AP-2prom604) which was generated for a previous study is given [15].

Interestingly, we now found that expression of the prominent hypoxia-sensitive gene* angiopoietin-like 4* (*ANGPTL4*) was also strongly induced during the hMSC spheroid differentiation and showed a striking correlation with expression of AP-2*ε* (Figure 1(a)). It is known that expression of ANGPTL4 in chondrocytes and other cell types is heavily dependent on low oxygen concentrations [29–31]. Hence, the expression pattern of ANGPTL4 indicated that oxygen supply to the cells within the spheroids vastly decreases during the differentiation progress and that this affects gene expression in these cells. Further, AP-2*ε* and Angptl4 also showed a similar expression pattern* in vivo *comparing early and late stages of cartilage development (Figure 1(b)). More precisely, we analyzed gene expression in condensed mesenchymal cells derived from the limb buds of E11.5 mouse embryos shortly prior to cartilage formation and in epiphyseal cartilage of new born miceharbouring highly differentiated chondrocytes [32]. In the latter, we detected a significant increase in the expression rate of both AP-2*ε* and the hypoxia marker Angptl4 (Figure 1(b)). As it was already shown that the reduction of oxygen supply is an important external stimulus contributing to limb and cartilage morphogenesis and due to the confirmatory results from the two independent systems, hypoxia could represent a trigger of AP-2*ε* expression throughout chondrocyte differentiation [12, 33]. To address this question, the human and the murine* AP-2ε* promoter sequences were analyzed for hypoxia responsive elements (HREs) (−2999 bp relative to the translation start). An alignment of the two sequences revealed that only the first 600 bp is generally well conserved across the two species (data not shown). In this region one HRE with the sequence 5′-CCGTG-3′ could be identified in both species (−108 to −104 bp in the human promoter and −89 to −85 bp in the murine promoter) (Figure 2). Additionally, in each species three more HRE motives were found further upstream within the promoter sequence (Figure 2). The presence of these putative HIF1 binding sites within the* AP-2ε* promoter in both species further supported the hypothesis of hypoxia being a general trigger of AP-2*ε* expression in chondrogenic cell types.

**Figure 3:**
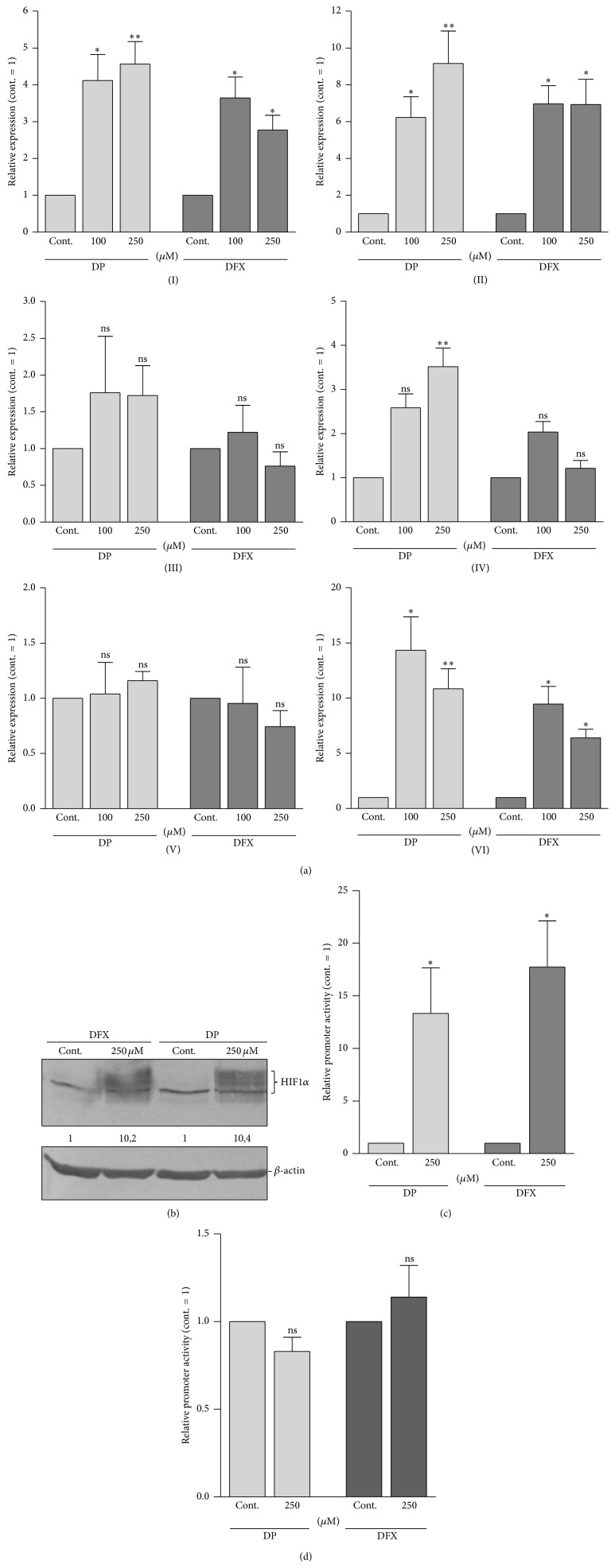
Upregulation of AP-2*ε* mRNA expression in mMSC after treatment with DP and DFX. (a) mMSC were treated with the hypoxia-mimicking iron chelators 2,2′-dipyridyl (DP) and desferrioxamine (DFX) at a concentration of 100 and 250 *μ*M. Respective controls were incubated with solvent only. 24 h later total RNA was isolated and mRNA expression was analyzed via qRT-PCR. Compared to the controls, a significant upregulation in the expression of AP-2*ε* (I) as well as of the positive control Angptl4 (II) was detected after DP/DFX treatment. Expression of Col2a1 (III) and Acan (IV) also tended to be enhanced while expression of MIA/CD-RAP (V) was completely unaffected by DP/DFX. Further, a significant induction of Sox9 (VI) expression could be determined. (b) HIF1*α* protein accumulation due to the chemical compounds was confirmed by western blot analysis. Numbers indicate densitometric measurement of the intensity of the HIF1*α* specific band (labeled). (c) Enhanced HIF1 transcriptional activity was shown by transfection of a reporter plasmid containing six consecutive HRE binding motifs (6xHRE) into mMSC which were treated as above. 24 h later luciferase activity was measured and a significant upregulation of promoter activity could be detected after incubation with DP/DFX. (d) Additionally, the activity of a 604 bp human* AP-2ε* promoter construct (AP-2prom604) was measured in the cells. DP/DFX treatment for 24 h did not result in an upregulation of its activity (data are given as means ± SEM; ns, not significant; ^*∗*^ *p* < 0.05; ^*∗∗*^ *p* < 0.01).

In order to investigate this, we performed experiments targeting intracellular activity of HIF1, the primary mediator of adaptive responses to hypoxia. First, we treated murine mesenchymal stem cells (mMSC) with the hypoxia-mimicking iron chelators 2,2′-dipyridyl (DP) and desferrioxamine (DFX) for 24 h at a concentration of 100 and 250 *μ*M, respectively. Compared to the respective controls, we detected a significant upregulation of AP-2*ε* mRNA expression in cells incubated with DP/DFX (Figure 3(a)I). The same result was obtained for the positive control Angptl4 (Figure 3(a)II). Additionally, expression of the prominent cartilage matrix proteins type 2 collagen (Col2a1), Aggrecan (Acan), and melanoma inhibitory activity/cartilage-derived retinoic acid-sensitive protein (MIA/CD-RAP) was analyzed. HIF1-dependent modulation of Col2a1 and Acan expression has already been described and also here expression of both tended to be enhanced albeit only partially statistically significant (Figure 3(a)III, IV) [34]. In contrast, the transcription rate of MIA/CD-RAP was completely unaffected by DP/DFX (Figure 3(a)V). Furthermore, in accordance with publications of other groups describing Sox9 as regulated by HIF1, a significant induction of Sox9 expression could be determined in this experiment (Figure 3(a)VI) [13, 35]. Intracellular HIF1*α* protein accumulation triggered by the chemical compounds was controlled by western blot analysis (Figure 3(b)). In addition, enhanced transcriptional activity of the HIF1 protein complex in the nuclei of the cells was confirmed with a luciferase reporter construct driven by a promoter fragment containing six hypoxia-responsive elements (6xHRE) from the human phosphoglycerate kinase gene. As expected, a strong upregulation of promoter activity could be measured after incubation with DP/DFX (Figure 3(c)). In one of our previous studies, a 604 bp human* AP-2ε* promoter construct (AP-2prom604; −604 to −1 bp relative to the translation start) in a luciferase reporter gene vector was generated [15]. As already described, the respective DNA region contains a HIF1 binding motive, which can also be found in the murine promoter (cf. Figure 2), in addition to the Sox9 binding site analyzed previously. Thus, the construct was used to test whether this motive could be responsible for the DP/DFX-mediated induction of AP-2*ε* mRNA expression. AP-2prom604 was active in the murine cells (approximately 3-fold relative to the control vector pGL3basic). However, DP/DFX treatment did not result in an upregulation of* AP-2ε* promoter activity, thereby excluding this HRE motive and hypoxia-dependent regulation of Sox9 to be regulatory active (Figure 3(d)).

**Figure 4:**
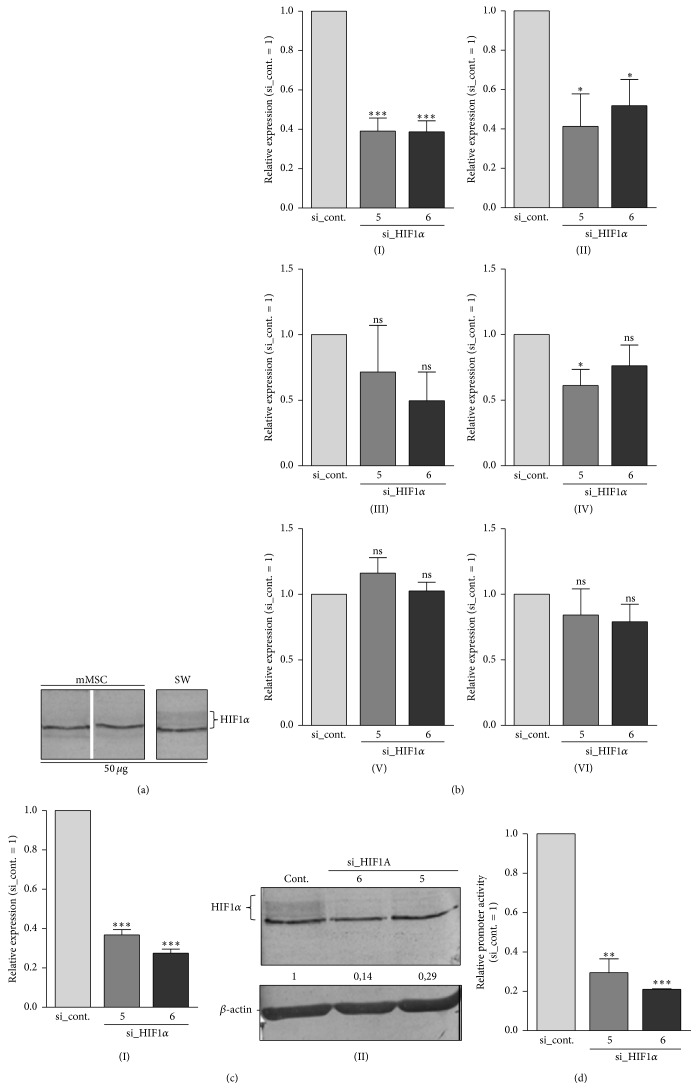
Downregulation of AP-2*ε* mRNA expression in SW1353 cells after treatment with siRNA against HIF1*α*. (a) HIF1*α* protein expression under normoxic conditions was compared in mMSC and SW1353 chondrosarcoma cells via western blot. SW1353 cells exhibited much higher physiological HIF1*α* protein levels (the HIF1*α* specific band is labeled; cf. Methods,* Protein Isolation and Western Blot*) and thus were preferred for the following HIF1*α* knockdown experiment. (b) mRNA expression was analyzed in SW1353 cells after transfection with two siRNA species against HIF1*α*. Compared to cells transfected with unspecific control siRNA, a significant reduction of the mRNA level of AP-2*ε* (I) and ANGPTL4 (II) could be determined. Expression of COL2A1 (III) and ACAN (IV) also tended to be reduced while expression of MIA/CD-RAP (V) and SOX9 (VI) was not significantly altered in this experiment. (c) Successful depletion of HIF1*α* after siRNA treatment was confirmed on mRNA (I) and protein level (II). Numbers indicate densitometric measurement of the intensity of the HIF1*α* specific band (labeled). (d) To confirm modulation of the transcriptional activity of the HIF1 protein complex the 6xHRE reporter plasmid (cf. Figure 3(c)) was transfected into SW1353 cells which were treated as above. 24 h later luciferase activity was measured and a significant downregulation of promoter activity could be detected with siRNA against HIF1*α* (data are given as means ± SEM; ns, not significant; ^*∗*^ *p* < 0.05; ^*∗∗*^ *p* < 0.01; ^*∗∗∗*^ *p* < 0.001).

In a next experiment, we applied two siRNA species against HIF1*α* to knock down the HIF1*α* mRNA and protein level. We did not use mMSC for the knockdown experiment as protein expression of HIF1*α* in mMSC was very low under normoxia (Figure 4(a)) and the cells exhibited only low transfection efficiency. As a result no sufficient knockdown of HIF1*α* via siRNA could be established in this cell type. Instead, we employed SW1353 chondrosarcoma cells for the application of siRNA against HIF1*α* because of their relatively high physiological HIF1*α* protein expression (Figure 4(a)). Here, a significant downregulation in the expression rate of AP-2*ε* as well as the positive control ANGPTL4 was detectable in cells transfected with siRNA against HIF1*α* (Figure 4(b)I, II). Further, as expected, expression of COL2A1 and ACAN tended to be reduced after the siRNA treatment while MIA/CD-RAP againstayed completely unchanged (Figure 4(b)III, IV, V). In contrast to the DP/DFX experiment, expression of SOX9 was not significantly altered by the HIF1*α* knockdown (Figure 4(b)IV). Successful knockdown of HIF1*α* was confirmed on mRNA and protein level (Figure 4(c)I, II) as well as in luciferase assays using the 6xHRE reporter plasmid (Figure 4(d)). In addition, activity of the 604 bp* AP-2ɛ* promoter construct was determined in these cells after si_HIF1*α* transfection. Again, no response could be detected which underscores that modulation of* AP-2ε* gene expression by HIF1 happens outside of this 604 bp region (data not shown). Further, SW1353 cells were also incubated with DP/DFX to confirm the upregulation of AP-2*ε* observed in mMSC in the previous experiment (cf. Figure 3(a)I). As expected, both compounds led to a clear induction of AP-2*ε* expression (approximately 4.5-fold with 250 *μ*M DP and 2.5-fold with 250 *μ*M DFX) in the cell line (data not shown).

**Figure 5:**
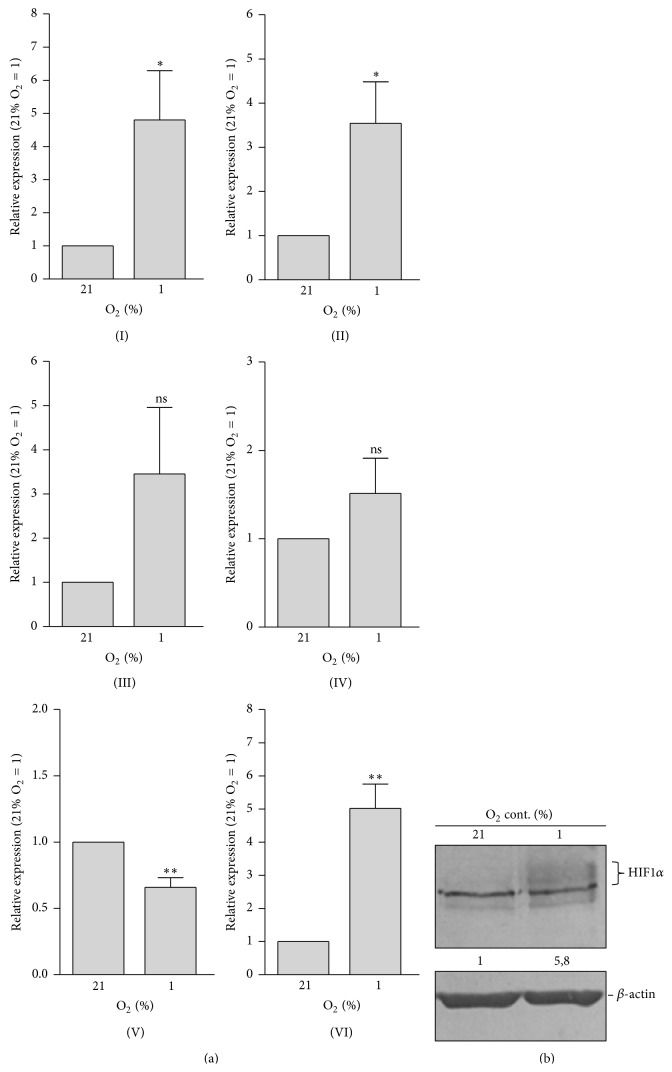
Upregulation of AP-2*ε* mRNA expression in response to hypoxia. (a) mMSC were cultured at hypoxia (1% O_2_) or normoxia (21% O_2_) for 24 h. After that, mRNA expression was analyzed via qRT-PCR. Compared to the control, a significant upregulation of AP-2*ε* (I) as well as Angptl4 (II) and Sox9 (IV) expression in cells incubated at 1% O_2_ was detected. Expression of Col2a1 (III) and Acan (IV) also tended to be enhanced while MIA/CD-RAP (V) was even slightly downregulated. (b) HIF1*α* protein accumulation due to hypoxia was confirmed by western blot analysis. Numbers indicate densitometric measurement of the intensity of the HIF1*α* specific band (labeled) (data are given as means ± SEM; ns, not significant; ^*∗*^ *p* < 0.05; ^*∗∗*^ *p* < 0.01).

Finally, we cultivated mMSC for 24 h under real hypoxia at low oxygen concentration (1% O_2_) and compared the gene expression pattern to cells exposed to normoxic conditions (21% O_2_). Here, we detected an upregulation of AP-2*ε* and Angptl4 as well as Sox9 mRNA expression in response to hypoxia (Figure 5(a)I, II, VI). Expressions of Col2a1 and Acan were not significantly altered but there was a trend towards induction for both (Figure 5(a)III, IV). This time, MIA/CD-RAP was slightly downregulated (Figure 5(a)V). HIF1*α* protein accumulation in response to hypoxia was controlled by western blot analysis (Figure 5(b)).

Taken together, our* in vitro* experiments clearly show that hypoxia plays an important role in the regulation of AP-2*ε*. Further, the HIF1*α* knockdown experiment confirms that the transcription factor complex HIF1 is a mediator of this effect. We feel that these results provide a reasonable explanation for the observed increase of AP-2*ε* expression during spheroid differentiation of human cells as well as during murine cartilage development.

Hence, the study underlines the important role of hypoxia as an external regulatory stimulus during chondrogenic differentiation influencing the expression of down-stream transcription factors like AP-2*ε*.

Our approaches revealed a novel role for the HIF1 complex in the regulation of the* AP-2ε* gene in cartilaginous cells and underlined the important role of hypoxia as an important external regulatory stimulus during chondrogenic differentiation modulating the expression of down-stream transcription factors.

## 4. **Discussion**

Previously, we discovered expression of AP-2*ε* in murine hypertrophic chondrocytes and in human articular cartilage. Additionally, an upregulation of AP-2*ε* transcript levels during late stages of chondrogenic differentiation of hMSC in 3D spheroid culture could be detected [15, 16]. We performed a similar experiment for this study and found that the expression of* ANGPTL4*, a prominent hypoxia marker and HIF1 target gene, was also strongly induced during the differentiation process indicating a definite decrease in the oxygen supply to the cells within the spheroids. This, most likely, is explained by increasing cell aggregation and compaction as well as progressive extracellular matrix production as a result of the chondrogenic differentiation process. The latter was confirmed by enhanced expression of the cartilage ECM proteins COL2a1, ACAN, and MIA/CD-RAP (cf. supplementary Figure 1, in Supplementary Material available online at http://dx.doi.org/10.1155/2015/380590). Presumably, these effects minimize diffusion of media and oxygen into the interior of the spheroids resulting in hypoxic conditions and thus upregulation of ANGPTL4 expression.

Another correlation between* Angptl4* and* AP-2ε* could be determined* in vivo *as expression of both genes was significantly upregulated in differentiated epiphyseal chondrocytes from new born mice compared to mesenchymal cells derived from embryonic limb buds (E11.5). It was shown that very early limb bud mesenchyme of mouse embryos (E10.5) is hypoxic exhibiting expression and transcriptional activity of HIF1*α* [33]. We assumed that hypoxia is also important for later stages of cartilage development because oxygen supply to chondroblasts/chondrocytes vastly decreases as the volume of the avascular cartilaginous template increases [36].

Due to these findings we hypothesized that hypoxia is an external stimulus regulating not only Angptl4 but also AP-2*ε* expression in chondrogenic cells and the current study was designed to investigate this in detail. In turn, the obtained data should help to enhance our understanding of mechanisms controlling gene expression in chondrocytes.

To define the influence of HIF1 and hypoxia on AP-2*ε* expression, we conducted three experimental approaches. Treatment of mMSC with the PHD inhibitors DP or DFX [9, 37] as well as cultivation of mMSC at 1% atmospheric O_2_ resulted in a significant upregulation of the* AP-2ε* transcription rate. Conversely, siRNA mediated downregulation of HIF1*α* in SW1353 cells significantly reduced AP-2*ε* expression. In sum, these three experiments clearly show that AP-2*ε* expression is regulated by hypoxia and that HIF1 is an intermediary factor involved in this process.

Interestingly, in the DP/DFX and 1% O_2_ experiments also an induction of Sox9 expression could be determined. It has already been reported that the gene encoding this transcription factor is a target of HIF1 and is upregulated in cartilaginous cells and tissues subjected to hypoxic conditions [13, 35]. Additionally, as mentioned in the introduction, we were able to show that SOX9 is a transactivator of the human* AP-2ε* gene in SW1353 cells [15]. Thus, Sox9/SOX9 could have represented a link between hypoxia and the induction of AP-2*ε* expression. However, several lines of evidence speak against this theory.

Firstly,* MIA/CD-RAP* was not and* Col2a1* was only slightly upregulated in the experiments despite both being directly activated by Sox9 (cf. Figures 3(a) and 5(a)) [38–40]. A possible explanation for this is that expression of Sox9 might be induced by DP/DFX and 1% O_2_ with a certain temporal delay which would explain why we did not yet observe a significant increase in MIA/CD-RAP and Col2a1 expression when the cells were harvested 24 hours after initiation of the treatment. However, AP-2*ε* expression already was strongly upregulated at this time indicating that this happens independently of Sox9.

Secondly, the activity of a human 604 bp* AP-2ε* promoter construct was not altered by DP/DFX in mesenchymal cells although in a previous study we could show that the respective DNA region contains a consensus SOX9 binding site (−450 to −445 bp), which mediates activation of* AP-2ε* transcription by SOX9 in SW1353 cells [15]. Hence, it is likely that Sox9 alone is not sufficient to trigger AP-2*ε* expression in this cell type in contrast to the SW1353 chondrosarcoma cell line, probably due to a missing coactivator in the mesenchymal cells. Contrary to the promoter fragment, AP-2*ε* mRNA expression was induced in the mMSC after DP/DFX treatment which indicates that this happens independently of Sox9.

Thirdly, in contrast to AP-2*ε* the expression of SOX9 was not significantly reduced after siRNA transfection against HIF1*α* in SW1353 cells. Currently, we do not know why* SOX9* was not stronger regulated in this assay; however, this finding again supports that* AP-2ε* induction in response to hypoxia is independent of SOX9.

In summary, AP-2*ε* is either a direct HIF1 target gene or indirectly regulated by means of an intermediate transcription factor other than Sox9/SOX9 under hypoxic conditions. Concerning the aforementioned, examination of the* AP-2ε* promoter sequence (−1 to −2999 bp relative to the translation start) revealed four putative HIF binding sites in humans and mice (cf. Figure 2). As already stated, the activity of the proximal −604 bp* AP-2ε* promoter construct was not altered after DP/DFX treatment of mMSC. The same was true for transfection of siRNA against HIF1*α* in SW1353 cells. Hence, it can be excluded that this region including its HRE motive mediates the upregulation of AP-2*ε* expression. However, in each species three further HRE motives can be found (cf. Figure 2). Although the positions are not directly conserved, one of them might represent the HIF1 binding site inducing promoter activity and* AP-2ε* gene expression. Further experiments are needed to define whether HIF1 indeed interacts with one of these motives or alternatively to investigate a potential indirect mode of regulation via an intermediate factor. Certainly, hypoxia-dependent regulation of AP-2*ε* expression in both human and murine cells suggests HIF1 to be a conserved mediator influencing* AP-2ε* gene transcription in mammals. Finally, it was reported that an additional depletion of oxygen supply is a hallmark of osteoarthritic cartilage accompanied by an increase of HIF1 transcriptional activity [41, 42]. This finding might be an explanation for the enhanced AP-2*ε* expression observed in osteoarthritic chondrocytes where it regulates the expression of the chemokine CXCL1, an inductor of ECM calcification and degradation [21].

## 5. **Conclusion**

Taken together, we discovered a significant increase in the expression rate of AP-2*ε* during chondrocyte differentiation* in vitro* and* in vivo* and our experiments provide evidence that hypoxia is crucial for this process. Further, we revealed a novel role for HIF1 as a direct or indirect transactivator of* AP-2ε* gene expression in chondrogenic cells. Thus, the study underscores hypoxia as an important external stimulus regulating down-stream gene expression during cartilage development.

## Supplementary Material

Supplementary Figure 1. Expression of matrix proteins during chondrogenic differentiation of hMSC spheroids 3-D spheroid cultures of hMSC were cultured in chondrogenic medium for a period of 28 days and mRNA was isolated at day 1, 14, 21 and 28. Expression of COL2A1 (I), ACAN (II) and MIA/CD-RAP (III) strongly increased over the course of the experiment confirming the chondrogenic differentiation process. (Data are given as means ± SEM).

## Figures and Tables

**Table 1:** Primer pairs used for quantitative real-time PCR.

Gene	Species	Primer	Product	Sequence (5′-3′)

*ACAN *	Human	*hAggrecan_6062for* *hAggrecan_6224rev *	162 bp	CCTACCAAGTGGCATAGC TGTTGGAGCCTGGGTTAC

*Acan *	Murine	*mAggrecan_1922for* *mAggrecan_2128rev *	206 bp	CAGTTCACCTTCCAGGAAG GTAGAGGTAGACCGTTCTCACG

*ACTB *	Human	*hβ-Act_735for* *hβ-Act_1119rev *	384 bp	CTACGTCGCCCTGGACTTCGAGC GATGGAGCCGCCGATCCACACGG

*Actb *	Murine	*mβ-Act_885for* *mβ-Act_1233rev *	348 bp	TGGAATCCTGTGGCATCCATGAAAC TAAAACGCAGCTCAGTAACAGTCCG

*ANGPTL4 *	Human	*hAngptl4_180for* *hAngptl4_354rev *	174 bp	CAGGGTACCTAAGAGGATGAGCGGTG CTGCTCGAGCTGCAGGAGTCCGTGC

*Angptl4 *	Murine	*mAngptl4_1036for* *mAngptl4_1304rev *	268 bp	GATGGCAATGCCAAATTGCTCC TGCCGTGGGATAGAGTGGAAG

*TFAP2E *	Human	*hAP-2ε_1690for* *hAP-2ε_1958rev *	268 bp	GGAGTAAGGGAGGGTGGCCTCTC GGGTGTCGCTGTTGAAGTCAGAGG

*Tfap2e *	Murine	*mAP-2ε_388for* *mAP-2ε_542rev *	154 bp	GCCGACCCTGGGGAGCTACAC CACCTCCGGCGCCGCTTAAA

*COL2A1 *	Human	*hCol2_4452for* *hCol2_4749rev *	297 bp	AGGGCAATAGCAGGTTCACG GGTCAGGTCAGCCATTCAGT

*Col2a1 *	Murine	*mCol2_2657for* *mCol2_2918rev *	262 bp	CTACTGGAGTGACTGGTCCTAAGG GGACCATCATCTCCAGGTTCTCC

*HIF1A *	Human	*hHif1α_866for* *hHif1α_1031rev *	165 bp	CACAGGCCACATTCACGTA ATCCAGGCTGTGTCGACTG

*MIA/CD-RAP *	Human	*hMia_220for* *hMia_544rev *	324 bp	CATGCATGCGGTCCTATGCCCAAGCTG GATAAGCTTTCACTGGCAGTAGAAATC

*MIA/CD-RAP *	Murine	*mMia_184for* *mMia_391rev *	207 bp	CCAAGCTGGCTGACTGGAAG GCCAGGTCTCCATAGTAACC

*SOX9 *	Human	*hSox9_1552for* *hSox9_1774rev *	222 bp	CGAACGCACATCAAGACGA AGGTGAAGGTGGAGTAGAGGC

*Sox9 *	Murine	*mSox9_799for* *mSox9_926rev *	127 bp	CTCTGGAGGCTGCTGAACGAGAGC TTCTTCACCGACTTCCTCCGCCG

## References

[1] Thorogood P. V., Hinchliffe J. R. (1975). An analysis of the condensation process during chondrogenesis in the embryonic chick hind limb. *Journal of Embryology and Experimental Morphology*.

[2] Karsenty G., Wagner E. F. (2002). Reaching a genetic and molecular understanding of skeletal development. *Developmental Cell*.

[3] Lefebvre V., Smits P. (2005). Transcriptional control of chondrocyte fate and differentiation. *Birth Defects Research Part C—Embryo Today*.

[4] Goldring M. B., Tsuchimochi K., Ijiri K. (2006). The control of chondrogenesis. *Journal of Cellular Biochemistry*.

[5] Karsenty G. (2008). Transcriptional control of skeletogenesis. *Annual Review of Genomics and Human Genetics*.

[6] Lefebvre V., Bhattaram P. (2010). Vertebrate skeletogenesis. *Current Topics in Developmental Biology*.

[7] Wang G. L., Jiang B.-H., Rue E. A., Semenza G. L. (1995). Hypoxia-inducible factor 1 is a basic-helix-loop-helix-PAS heterodimer regulated by cellular O2 tension. *Proceedings of the National Academy of Sciences of the United States of America*.

[8] Kallio P. J., Pongratz I., Gradin K., McGuire J., Poellinger L. (1997). Activation of hypoxia-inducible factor 1*α*: posttranscriptional regulation and conformational change by recruitment of the Arnt transcription factor. *Proceedings of the National Academy of Sciences of the United States of America*.

[9] Kallio P. J., Wilson W. J., O'Brien S., Makino Y., Poellinger L. (1999). Regulation of the hypoxia-inducible transcription factor 1*α* by the ubiquitin-proteasome pathway. *The Journal of Biological Chemistry*.

[10] Masson N., Ratcliffe P. J. (2003). HIF prolyl and asparaginyl hydroxylases in the biological response to intracellular O_2_ levels. *Journal of Cell Science*.

[11] Ke Q., Costa M. (2006). Hypoxia-inducible factor-1 (HIF-1). *Molecular Pharmacology*.

[12] Schipani E. (2005). Hypoxia and HIF-1*α* in chondrogenesis. *Seminars in Cell and Developmental Biology*.

[13] Amarilio R., Viukov S. V., Sharir A., Eshkar-Oren I., Johnson R. S., Zelzer E. (2007). HIF1*α* regulation of Sox9 in necessary to maintain differentiation of hypoxic prechondrogenic cells during early skeletogenesis. *Development*.

[14] Schipani E., Ryan H. E., Didrickson S., Kobayashi T., Knight M., Johnson R. S. (2001). Hypoxia in cartilage: HIF-1*α* is essential for chondrocyte growth arrest and survival. *Genes & Development*.

[15] Wenke A.-K., Grässel S., Moser M., Bosserhoff A. K. (2009). The cartilage-specific transcription factor Sox9 regulates AP-2*ε* expression in chondrocytes. *FEBS Journal*.

[16] Wenke A.-K., Rothhammer T., Moser M., Bosserhoff A. K. (2006). Regulation of integrin *α*10 expression in chondrocytes by the transcription factors AP-2*ε* and Ets-1. *Biochemical and Biophysical Research Communications*.

[17] Feng W., Williams T. (2003). Cloning and characterization of the mouse AP-2 epsilon gene: a novel family member expressed in the developing olfactory bulb. *Molecular and Cellular Neuroscience*.

[18] Tummala R., Romano R.-A., Fuchs E., Sinha S. (2003). Molecular cloning and characterization of AP-2*ε*, a fifth member of the AP-2 family. *Gene*.

[19] Hilger-Eversheim K., Moser M., Schorle H., Buettner R. (2000). Regulatory roles of AP-2 transcription factors in vertebrate development, apoptosis and cell-cycle control. *Gene*.

[20] Huang Z., Xu H., Sandell L. (2004). Negative regulation of chondrocyte differentiation by transcription factor AP-2*α*. *Journal of Bone and Mineral Research*.

[21] Wenke A.-K., Niebler S., Grässel S., Bosserhoff A. K. (2011). The transcription factor AP-2ɛ regulates CXCL1 during cartilage development and in osteoarthritis. *Osteoarthritis and Cartilage*.

[22] Weston A. D., Rosen V., Chandraratna R. A. S., Underhill T. M. (2000). Regulation of skeletal progenitor differentiation by the BMP and retinoid signaling pathways. *The Journal of Cell Biology*.

[23] James C. G., Appleton C. T. G., Ulici V., Michael Underhill T., Beier F. (2005). Microarray analyses of gene expression during chondrocyte differentiation identifies novel regulators of hypertrophy. *Molecular Biology of the Cell*.

[24] Stanton L.-A., Beier F. (2007). Inhibition of p38 MAPK signaling in chondrocyte cultures results in enhanced osteogenic differentiation of perichondral cells. *Experimental Cell Research*.

[25] Schmid R., Schiffner S., Opolka A. (2010). Enhanced cartilage regeneration in MIA/CD-RAP deficient mice. *Cell Death and Disease*.

[26] Johnstone B., Hering T. M., Caplan A. I., Goldberg V. M., Yoo J. U. (1998). In vitro chondrogenesis of bone marrow-derived mesenchymal progenitor cells. *Experimental Cell Research*.

[27] Yoo J. U., Barthel T. S., Nishimura K. (1998). The chondrogenic potential of human bone-marrow-derived mesenchymal progenitor cells. *The Journal of Bone and Joint Surgery—American Volume*.

[28] Klinger P., Schietke R. E., Warnecke C. (2011). Deletion of the oxygen-dependent degradation domain results in impaired transcriptional activity of hypoxia-inducible factors. *Transcription*.

[29] Murata M., Yudo K., Nakamura H. (2009). Hypoxia upregulates the expression of angiopoietin-like-4 in human articular chondrocytes: Role of angiopoietin-like-4 in the expression of matrix metalloproteinases and cartilage degradation. *Journal of Orthopaedic Research*.

[30] Zhang H., Wong C. C. L., Wei H. (2012). HIF-1-dependent expression of angiopoietin-like 4 and L1CAM mediates vascular metastasis of hypoxic breast cancer cells to the lungs. *Oncogene*.

[31] Zhu P., Goh Y. Y., Chin H. F. A., Kersten S., Tan N. S. (2012). Angiopoietin-like 4: a decade of research. *Bioscience Reports*.

[32] Wanek N., Muneoka K., Holler-Dinsmore G., Burton R., Bryant S. V. (1989). A staging system for mouse limb development. *Journal of Experimental Zoology*.

[33] Provot S., Zinyk D., Gunes Y. (2007). Hif-1alpha regulates differentiation of limb bud mesenchyme and joint development. *The Journal of Cell Biology*.

[34] Duval E., Leclercq S., Elissalde J.-M., Demoor M., Galéra P., Boumédiene K. (2009). Hypoxia-inducible factor 1*α* inhibits the fibroblast-like markers type I and type III collagen during hypoxia-induced chondrocyte redifferentiation: hypoxia not only induces type II collagen and aggrecan, but it also inhibits type I and type III collagen in the hypoxia-inducible factor 1*α*–dependent redifferentiation of chondrocytes. *Arthritis and Rheumatism*.

[35] Zhang C., Yang F., Cornelia R., Tang W., Swisher S., Kim H. (2011). Hypoxia-inducible factor-1 is a positive regulator of Sox9 activity in femoral head osteonecrosis. *Bone*.

[36] Bhosale A. M., Richardson J. B. (2008). Articular cartilage: structure, injuries and review of management. *British Medical Bulletin*.

[37] Woo K. J., Lee T.-J., Park J.-W., Kwon T. K. (2006). Desferrioxamine, an iron chelator, enhances HIF-1*α* accumulation via cyclooxygenase-2 signaling pathway. *Biochemical and Biophysical Research Communications*.

[38] Bell D. M., Leung K. K. H., Wheatley S. C. (1997). SOX9 directly regulates the type-II collagen gene. *Nature Genetics*.

[39] Lefebvre V., Huang W., Harley V. R., Goodfellow P. N., de Crombrugghe B. (1997). SOX9 is a potent activator of the chondrocyte-specific enhancer of the pro*α*1(II) collagen gene. *Molecular & Cellular Biology*.

[40] Xie W.-F., Zhang X., Sakano S., Lefebvre V., Sandell L. J. (1999). Trans-activation of the mouse cartilage-derived retinoic acid-sensitive protein gene by Sox9. *Journal of Bone and Mineral Research*.

[41] Yudoh K., Nakamura H., Masuko-Hongo K., Kato T., Nishioka K. (2005). Catabolic stress induces expression of hypoxia-inducible factor (HIF)-1 alpha in articular chondrocytes: involvement of HIF-1 alpha in the pathogenesis of osteoarthritis. *Arthritis Research & Therapy*.

[42] Pfander D., Gelse K. (2007). Hypoxia and osteoarthritis: how chondrocytes survive hypoxic environments. *Current Opinion in Rheumatology*.

