# Supplementary material for: Hypoxia-Inducible Factor 1 Is an Inductor of Transcription Factor Activating Protein 2 Epsilon Expression during Chondrogenic Differentiation
Source: Biomed Res Int. 2015 Jul 27;2015:380590. doi: 10.1155/2015/380590 (PMC4530219; doi:10.1155/2015/380590)
Supplement: Supplementary file 1 — Supplementary Figure 1. Expression of matrix proteins during chondrogenic differentiation of hMSC spheroids 3-D spheroid cultures of hMSC were cultured in chondrogenic medium for a period of 28 days and mRNA was isolated at day 1, 14, 21 and 28. Expression of COL2A1 (I), ACAN (II) and MIA/CD-RAP (III) strongly increased over the course of the experiment confirming the chondrogenic differentiation process. (Data are given as means ± SEM). [file 380590.f1.docx]

III

II

I

**Supplementary figure 1**
